# Glass Microdroplet Generator for Lipid-Based Double Emulsion Production

**DOI:** 10.3390/mi15040500

**Published:** 2024-04-05

**Authors:** Alessandra Zizzari, Valentina Arima

**Affiliations:** CNR NANOTEC-Institute of Nanotechnology c/o Campus Ecotekne, via Monteroni, 73100 Lecce, Italy; valentina.arima@nanotec.cnr.it

**Keywords:** microfluidics, glass device, double emulsions, lipid vesicles

## Abstract

Microfluidics offers a highly controlled and reproducible route to synthesize lipid vesicles. In recent years, several microfluidic approaches have been introduced for this purpose, but double emulsions, such as Water-in-Oil-in-Water (W/O/W) droplets, are preferable to produce giant vesicles that are able to maximize material encapsulation. Flow focusing (FF) is a technique used to generate double emulsion droplets with high monodispersity, a controllable size, and good robustness. Many researchers use polydimethylsiloxane as a substrate material to fabricate microdroplet generators, but it has some limitations due to its hydrophobicity, incompatibility with organic solvents, and the molecular adsorption on the microchannel walls. Thus, specific surface modification and functionalization steps, which are uncomfortable to perform in closed microchannels, are required to overcome these shortcomings. Here, we propose glass as a material to produce a chip with a six-inlet junction geometry. The peculiar geometry and the glass physicochemical properties allow for W/O/W droplet formation without introducing microchannel wall functionalization and using a variety of reagents and organic solvents. The robust glass chip can be easily cleaned and used repeatedly, bringing advantages in terms of cost and reproducibility in emulsion preparation.

## 1. Introduction

Double emulsions have received great interest in the scientific community, with a broad variety of applications in several fields of industry, such as food, cosmetic, and pharmaceutical products [1,2]. In particular, Water-in-Oil-in-Water (W/O/W) double emulsion droplets are valuable intermediate products that can generate giant lipid vesicles (GVs) that, in comparison to nanovesicles [3], are able to maximize material encapsulation, thus having more potential applications for cell models, biosensors, pharmaceutics, and foods [4,5,6,7,8,9].

The traditional methods used to produce double emulsions are bulk processes, such as sonication or high shear mixing [10]. Generally, the inner emulsion is formed in the first step, and then it is distributed in an external phase. The limits of these approaches are the low batch-to-batch reproducibility, the polydispersity of the double emulsion solutions, the production of undesired single emulsions, and a low encapsulation efficiency [11]. Droplet microfluidics can overcome these disadvantages, offering precise control over the size and morphology given that the droplet size depends on the shear force at the liquid–liquid interface and on the geometry of the microchannels [12]. But if the generation of monodisperse emulsions is a well-defined procedure, microfluidic double emulsion formation is still challenging. Shear-based microfluidic devices are valuable tools used to produce double emulsions, and the geometries for this purpose are double flow-focusing channels, serial T-junctions, and connected circular capillaries [7,13].

Besides geometry, the choice of material for the device’s fabrication is crucial for an efficient emulsion preparation. Microfluidic channels can be produced with several materials, such as silicon, glass, polymers, paper, and others [14]; however, glass and polydimethylsiloxane (PDMS) are the most used in droplet microfluidics [15]. PDMS-based devices emerge as the most suitable options due to their easy and cost-effective reproduction using a well-established soft lithography process [16,17], but surface treatments are required for internal channel walls because the intrinsic hydrophobicity of PDMS [11] as well as its marked tendency to adsorb molecules onto the microchannel’s wall surfaces are drawbacks for the production of W/O/W emulsions [16,18,19]. Additional limitations are PDMS’s susceptibility to swelling by organic solvents [20,21] and the leaching of small molecules from the PDMS bulk into solutions [22], which can obviously result in contamination or emulsion destabilization problems [13]. Therefore, although research into the modification of the PDMS surface is continuously developing, glass devices are tendentially preferred, albeit relatively expensive. In fact, thanks to its superior optical transparency, water wettability, mechanical stability, physicochemical properties, and resistance to solvents [14], glass is considered as the most convenient material for the fabrication of microfluidic devices [23].

**Figure 1:**
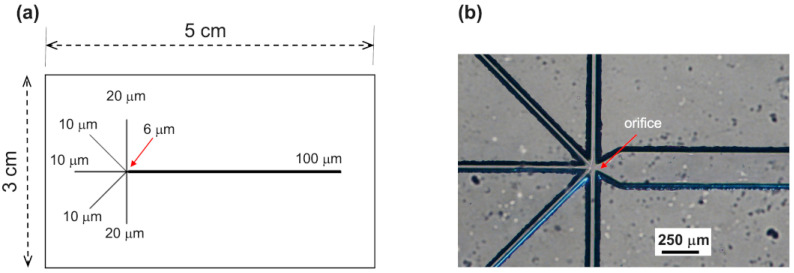
(**a**) The device scheme with a single six-channel FF junction. The reported measurements are the nominal microchannels’ widths; 6 μm is the orifice nominal width. (**b**) The optical image of the junction area where the orifice, indicated by the red arrow, is clearly visible.

**Figure 2:**
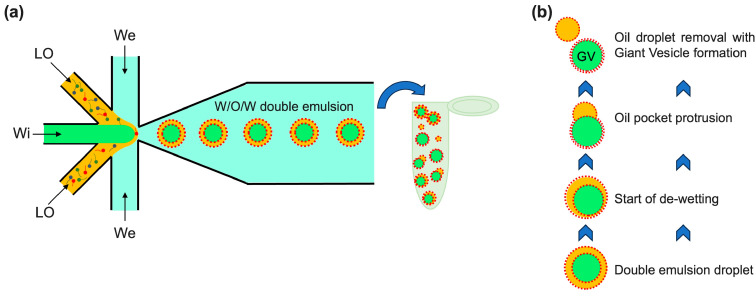
(**a**) A scheme of double emulsion droplet production and collection in a vial where the de-wetting process takes place. We, LO, and Wi are the external aqueous, oil with lipids, and internal aqueous phases, respectively. (**b**) De-wetting steps: from double emulsion to giant vesicle (GV) generation.

In this work, we used glass to fabricate a flow focusing device (FF) with a geometry that allows for double hydrodynamic FF (six-channel junction shown in Figure 1a,b) that is able to produce double emulsion (water/octanol–lipid/water) droplets in a single step. Each droplet consists of a water core directly encapsulated in an octanol shell containing lipids that, as already demonstrated [24], form a bilayer after organic solvent de-wetting and, therefore, a giant vesicle (see scheme in Figure 2a). The high hydrophilicity of glass microchannel walls makes our device suitable for producing (W/O/W) double emulsions in which the external continuous phase is water. Moreover, the physicochemical properties of the glass prevent the adsorption of liposomes in the post-junction area, so no surface treatment or coating is necessary as in the case of PDMS microchannels [13,25,26].

## 2. Materials and Methods

### 2.1. Solutions Preparation

To prepare the solutions, 1,2-Dioleoyl-sn-glycero-3-phosphocholine (DOPC) as solutions in chloroform and 1,2-dioleoyl-sn-glycero-3-phospho-rac-(1-glycerol) sodium salt (DOPG) were purchased from Avanti Polar Lipids. Texas Red 1,2-dihexadecanoyl-sn-glycero-3-phosphoethanolamine, triethylammonium salt (Texas Red-DHPE), and calcein (>93%) were purchased from Thermo Fisher Scientific (Monza, Italy). Chloroform, 1-octanol, and glycerol were acquired from Honeywell (Milan, Italy), and sucrose ultrapure was obtained from VWR-Life Science. Milli-Q water obtained using a purification system (Fulltech Instruments, Rome, Italy) was used for the preparation of all aqueous solutions.

The lipid mixture (DOPC:DOPG in a 1:1 ratio with 0.1 mol% Texas Red-DHPE) was dissolved in chloroform in a glass vial. Chloroform was evaporated under a gentle stream of nitrogen, and the lipids were further dried by desiccating for at least 1 h. After adding octanol in order to achieve a final lipid concentration of 15 mg/mL, the lipid–oil solution (LO) was sonicated for 30 min and then filtered using 0.45 µm syringe filters. The internal aqueous solution (Wi) was 600 mM sucrose with 0.05 mM calcein, and the external aqueous solution (We) was 600 mM glucose.

### 2.2. Glass Device Fabrication and Pumping Set-Up

To fabricate the device, commercially available B-270 glasses covered with a 450 nm thick chromium layer (Telic) were used as solid substrates. Hydrochloric acid (HCl), ammonium fluoride (NH4F), and hydrofluoric acid (HF) were purchased from Sigma-Aldrich (Taufkirchen, Germany). The resist AZ10XT and the AZ400k developer were purchased from MicroChemicals (Ulm, Germany). The chromium etchant solution was purchased from Sigma-Aldrich. Photomasks were designed using CleWin software (Version 2.67) and printed by J. D. Photo-tools Ltd. (Oldham, Lancashire, UK).

The microfluidic pattern reported In the scheme of Figure 1a was obtained by combining the techniques of optical lithography and wet etching. A layer of AZ10XT was deposited by spin coating on a B-270 glass substrate. This layer was then processed by UV exposure by using a photomask to transfer the desired network of Figure 1a from the mask to the resist. After the selective remotion of the resist and chromium layer, the pattern on the glass was etched in a buffered oxide etchant solution to achieve microchannels about 30 μm deep. The wet etching was carried out in a microwave (Anton Paar Multiwave 3000, Labservice Analytica s.r.l., Bologna, Italy) as reported in ref. [27]. Then, six holes were made in correspondence with the inlets and outlets of the channels by means of a microdriller (MF70, Proxxon Micro miller, Föhren, Germany), and the patterned substrate was thermally bonded to a flat B-270 glass [28]. Successively, capillary tubes (Tub FEP Blu 1/32 × 0.09, IDEX Health & Science, Oak Harbor, WA, USA) were connected to the inlet and outlet holes.

The solutions prepared as described in Section 2.1 were flowed in capillary tubes and then in microchannels by using three mechanical syringe pumps (Ugo Basile, Biological Research Apparatus, model KDS270) to independently control the three phases’ (We, LO, and Wi) flow rates. To protect the operators from inhaling octanol vapor, the experiments were performed under a fume hood, and the fluids’ behaviors in the microchannels were observed by using a low-resolution camera. Then, the collected droplet solutions were observed and analyzed using an optical microscope (Nikon Eclipse Ti), and their images were acquired using a NIKON mod. DS-5MC camera.

## 3. Results and Discussion

We produced a glass microfluidic device that uses microdroplet technology to create W/O/W double emulsion droplets with an inner aqueous solution sheared by lipids dissolved in an organic solvent in a continuous aqueous phase (see scheme in Figure 2a). Then, giant lipid vesicles (GVs) were obtained from the double emulsions by spontaneously removing the oil from the organic layer of the W/O/W emulsions (see scheme in Figure 2b).

### 3.1. Chip Design

**Figure 3:**
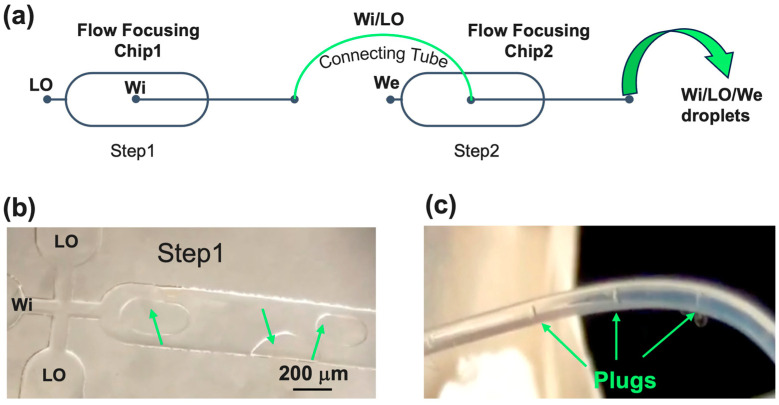
A “tandem system” device. (**a**) The sketch process: Two flow focusing chips were connected using a microtube. In the first droplet generator (Chip1), Step1 takes place, and Water-in-Oil (Wi/LO) droplets are produced. These droplets are the inputs of Step2 in the second droplet generator (Chip2), where they are sheared by the outer aqueous phase (We), thus generating double emulsions. (**b**) A glass “tandem system”: Without an adequate functionalization of the post-junction area, the Wi interacts with the hydrophilic walls, thereby resulting in the formation of large plugs indicated by green arrows. (**c**) The large plugs are visible in the connecting tube and, since they are inlets of the second chip, they compromise the success of the whole double emulsion formation process.

For chip design, two crucial aspects to be addressed are the microchannels’ geometry and chip fabrication material. The geometry of our microfluidic device includes six channels that cross at a single junction (Figure 1): one for Wi, two for LO, two for We, and one for the outlet. After the junction, of course, a downstream channel collects and flows the formed droplets. Through this configuration, our device allowed us to generate stable double emulsion droplets in a single step, making the process more user-friendly compared to existing microfluidic networks such as the so-called “tandem systems”, which are composed of two FF single junctions (Chip1 for Step1 and Chip2 for Step2) connected via tubing [29] (see scheme in Figure 3a), or geometries consisting of two consecutive FF single junctions performing these two steps in the same chip [30].

In both cases, synchronizing the two droplet generators is difficult, and double emulsion drops often result in a single shell with multiple cores whose number is hard to control [7]. In both layouts, Water-in-Oil (W/O) droplets can be produced in Step1 through the surface modification of the post-junction channel walls to ensure a contact angle greater than 120° [31]. In fact, as we also demonstrated (see Figure 3b,c), if the chip material is glass without an adequate functionalization of the post-junction area, it is difficult to generate stable W/O droplets from Step1 since, at the orifice level, where lipids start to assemble and act as stabilizers of the water/oil interface, the water phase may still interact with the hydrophilic walls. This results in the formation of large plugs in the output channel and tube from Chip1 which, being the input of Chip 2, compromise the success of the whole process of double emulsion formations (see Figure 3b). To overcome this problem, an additional step in the chip manufacturing is required to make the first outlet microchannel hydrophobic. If the chip is in PDMS, a surface treatment would still be needed because of its several limitations, as discussed hereafter [32]. Emulsion instability can also arise from flow alterations due to variable cross sections at the Chip1/tube/Chip2 connection (see Figure 3c). In addition, during the transport from the first emulsifier to the second one, the probability of weakly stabilized W/O droplets colliding increases as well as their coalescence due to collisions.

Some drawbacks due to the “tandem system” can be avoided using two consecutive FF junctions in the same chip, although this may always cause wettability issues for the channel walls. Indeed, to produce stable W/O/W double emulsions, hydrophobic walls are required in the first post-junction area to form W/O emulsions, and hydrophilic walls are required in the second post-junction area to generate W/O/W double emulsions from the W/O single emulsions.

Our design with six channels implemented in a single cross-junction can overcome many of these drawbacks. The Wi flow and the surrounding LO flows were hydrodynamically focused in the single six-channel junction and subsequently broken up by the two We streams. As soon as the W/O is formed, it is thereby sheared by the outer aqueous phase (see scheme of Figure 2b). In this way, there are no undesired section changes, and to generate stable W/O/W emulsions, the only requirement is that the post-area junction needs to be hydrophilic.

The fabrication material is crucial for the physicochemical properties of the internal channel walls, particularly for their hydrophobicity/hydrophilicity. Experts in microfluidics usually use PDMS or glass microcapillary. Unfortunately, due to the limitations described in the Introduction Section, such as hydrophobicity, the incompatibility between many organic solvents, and the adsorption of molecules (lipids in our case), PDMS requires pre-coating procedures that are often complicated and time-consuming. Indeed, to ensure robust coatings, multiple syringe pumps, elevated temperatures, or long incubation times are necessary [33,34]. These laborious procedures require experience in microfluidic techniques and the employment of reagents and set-up at additional costs. Moreover, coatings are not always stable over time, undergoing leaching or detachment, as well as in some operation conditions (i.e., pH condition changes [35]). To avoid all of these drawbacks of PDMS, a joint choice is the use of glass microcapillaries, which are very difficult to assemble due to the precise alignment steps [35].

In this work, we deployed glass to fabricate our device by combining the techniques of photolithography, wet etching, and thermal bonding to achieve closed microchannels as described in the Materials and Method Section. As an innovative approach, we take advantage of the natural glass properties of chemical inertness and hydrophilicity to avoid the pre-coating steps typical of the most common FF devices for double emulsion preparation reported in the literature. We demonstrate that the surface energies at the glass–lipids and glass–water interfaces do not impact the droplet generation process. By playing with the main flow parameters, stable double emulsions with an aqueous solution as an external phase can be produced without wasting time in the post-fabrication steps of surface functionalization. The process of droplet generation is entirely driven by interfacial energies and is not influenced by surface properties, meaning it is almost independent of the device’s material [36]. As a practical outcome, we could use our device several times since the glass can be easily cleaned, and we could use it for prolonged periods of time without problems of coating damage. Furthermore, in the absence of coatings that could be eroded by organic solvents [35], the fluids in our chips do not need to be injected into a precise order (such as to first flush the We before introducing the LO in the PDMS chip). This represents a practical advantage for operators dealing with microfluidic devices.

### 3.2. Flow Conditions for Generation of W/O/W Double Emulsions

To successfully generate double emulsions, octanol was chosen as an organic phase carrying lipids because it accomplishes a right balance between the poor miscibility with water and biocompatibility for applications in the biological field [25]. Furthermore, octanol is not only the ideal solvent due to these reasons, but also because it can quicken the de-wetting process by which the droplets spontaneously split into oil droplets and GVs after the formation of double emulsion droplets. This is called the OLA method, that is, octanol-assisted liposome assembly [37].

We used DOPC and DOPG as lipids that provide negative charges to the double emulsion droplets/GVs, preventing their fusion and enhancing their stability [24]. The mixture of these lipids was prepared with a molar ratio of 1:1 and a concentration of 15 mg/mL in octanol that is high enough not to require surfactants to stabilize the emulsions and to quicken the spontaneous de-wetting process [24]. The internal aqueous phase was 600 mM of sucrose solution, and the external one was 600 mM of glucose solution. To allow the imaging of double emulsion, 0.1 mol% of a fluorescent-labeled lipid (Texas Red-DHPE) was also added to the DOPC–DOPG mixture to view the shell, while 0.05 mM of calcein (green fluorescence) was added to Wi to view the core and to demonstrate the encapsulation efficiency of the approach.

**Figure 4:**
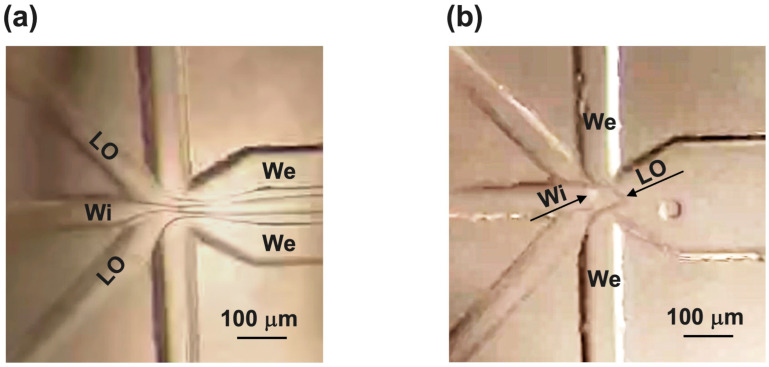
Fluids’ behaviors in microchannels observed by using low-resolution camera. (**a**) Co-flow work conditions (Wi = 5 μL/min, LO = 5 μL/min, and We = 50 μL/min). (**b**) Flow rate conditions for double emulsion droplet generation (Wi = 0.1 μL/min, LO = 0.1 μL/min, and We = 4 μL/min). Black arrows indicate Wi and LO phases’ meniscus. Oil phase enveloping Wi is sheared into droplets by We.

After some attempts with flow rates set at tens of μL/min for We and some μL/min for LO and Wi, resulting in co-flow conditions (see Figure 4a) without observing any fluids break up, we found the following conditions to be optimal to form double emulsion droplets: Wi = 0.1 μL/min, LO = 0.1 μL/min for each inlet, and We = 4 μL/min for each inlet. In the optical images of Figure 4b, we can see the oil phase enveloping Wi is sheared into droplets by We at the six-channel FF junction.

### 3.3. Off-Chip Characterization of W/O/W Double Emulsions

**Figure 5:**
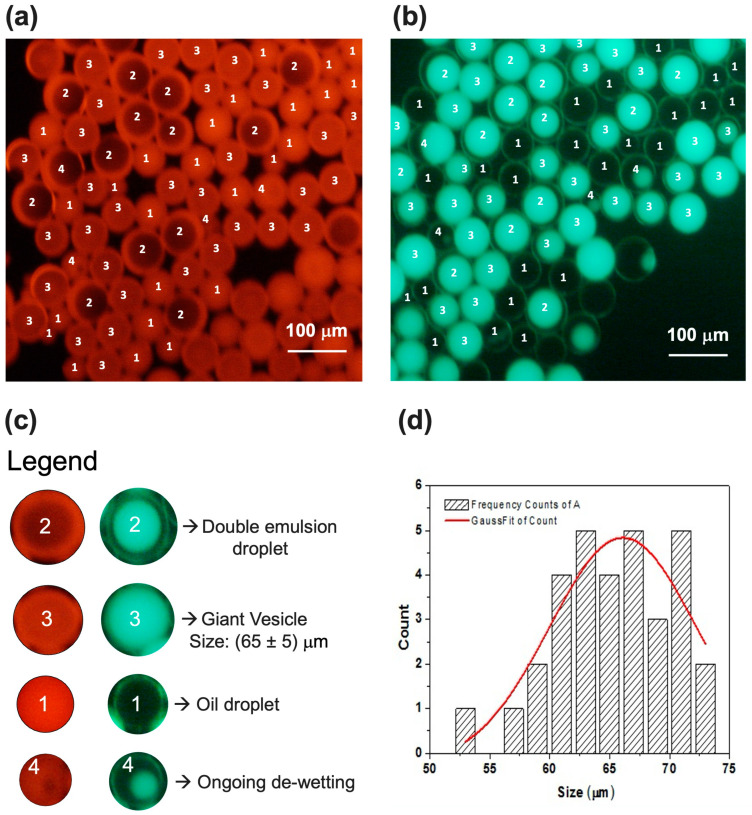
Fluorescence images of sample produced at Wi = 0.1 μL/min, LO = 0.1 μL/min, and We = 4 μL/min. Images were acquired under TRITC filter (**a**) and FITC filter (**b**). Both images show mixture of oil droplet (1), double emulsion droplet (2), GV (3), and partially de-wetting droplet (4). Legend is reported in (**c**). (**d**) GVs’ size distribution was obtained from a statistical analysis on several samples produced under same experimental conditions.

In Figure 5, the optical characterization of a batch solution resulting from these flow rate conditions is reported. The double emulsion droplets are clearly visible in Figure 5a. The red shells (ring structures) correspond to the fluorescent octanol–lipid layers, whereas the green fluorescence is in correspondence with calcein in the aqueous cores. In Figure 5b, we can see thin green shells, which occur simply due to the lensing effect of the calcein fluorescence from the background solution [24]. The de-wetting process was already started since, in addition to the double emulsion droplets, the collected sample showed GVs and partially de-wetted double emulsion droplets with oil droplets still attached. The red fluorescence of GVs is homogeneous compared to the ring structure shown by the double emulsion droplets. The size of these vesicles is 65 ± 5 μm, as calculated in Figure 5d. Although we used a lipid concentration of 15 mg/mL and similar flow rates, our sample not only showed vesicles, but also double emulsion droplets compared to what was reported by Bao et al. We could attribute the different behavior to the filtration we performed on the lipid solutions before injection in the chip as well as to a different interaction between the PDMS and glass walls with the lipid molecules. Both of these phenomena may impact the real lipid concentration and, consequently, the de-wetting process that is strongly concentration-dependent.

**Figure 6:**
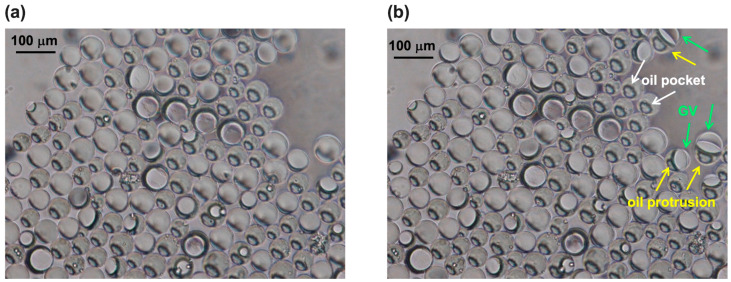
Optical images of the de-wetting phenomenon acquired at different times are shown in (**a**,**b**). The starting step is the formation of an oil pocket inside the double emulsion (some are indicated with white arrows), and then the oil drop protrudes (some are indicated with yellow arrows) until its final detachment, thus generating a giant vesicle (GV) (some are indicated with green arrows).

In the optical image of Figure 6a,b, the de-wetting process is clearly visible. Upon the depletion force generated by excess lipid molecules in the solvent, the separation of octanol from double emulsion drops started [33] so that the excess organic solvent accumulated in a pocket inside the double emulsion droplet (indicated by white arrows in Figure 6b). Then, the oil pocket started to protrude outwards, as indicated by the yellow arrows in Figure 6b, and the Wi droplet moved across the interface O/We, while the lipids at the We/O interface stabilized the escaping Wi droplet by completing the bilayers (see Scheme in Figure 2b). At this point, the oil droplet completely budded off, and the lipids, which were initially concentrated and arranged on the double emulsion interface, formed the GVs.

**Figure 7:**
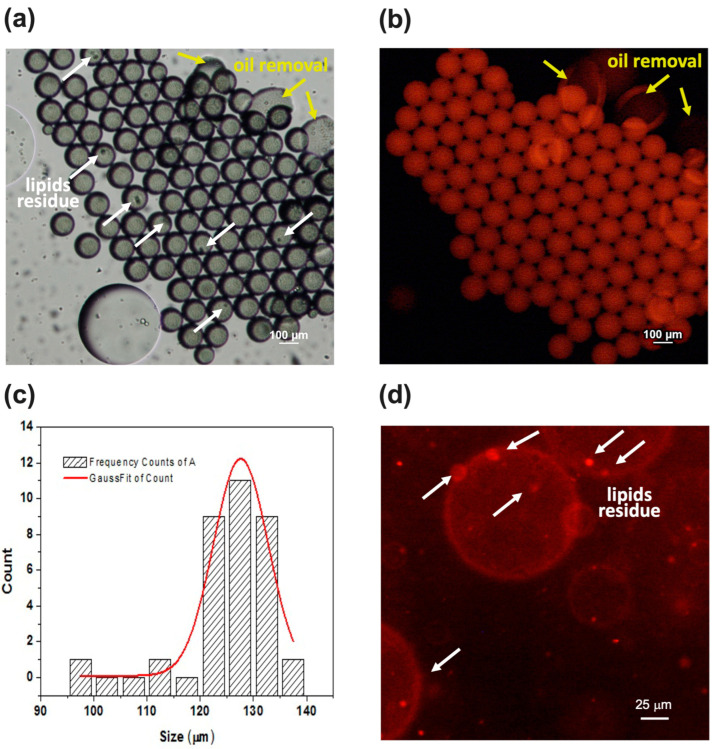
Images of sample produced at Wi = 1 μL/min, LO = 0.1 μL/min, and We = 1.5 μL/min. Images show only GVs since complete de-wetting took place for almost all double emulsion droplets. (**a**) Optical image of vesicles: oil with lipid residue remains inside some GVs. (**b**) Image of GVs acquired under TRITC filter. (**c**) GV size distribution. (**d**) GVs are quite stable even after five days, as shown in red fluorescence image. Here, lipid residues attached to GVs are clearly visible.

We produced another sample by changing the flow rate conditions, which were Wi = 1 μL/min, LO = 0.1 μL/min, and We = 1.5 μL/min for each inlet. As shown in Figure 7a,c, the batch resulted in only GVs, thus showing a complete de-wetting, which was probably due to a decrease in the shell thickness. Indeed, with a thinner shell, the amount of octanol was reduced, and faster removal occurred [35]. The reduction in the thickness of the shell was probably a consequence of a higher flow rate ratio between Wi (1 μL/min) and LO (0.1 μL/min) with respect to the previous batch obtained by setting both the Wi and LO at 0.1 μL/min. On the contrary, the lower flow rate ratio between We (1.5 μL/min) and LO (0.1 μL/min), with respect to the previous batch (We = 4 μL/min and LO = 0.1 μL/min), led to an increase in size (126 ± 7 μm).

In the bright field image of Figure 7a, a dark spot is visible inside some vesicles. It corresponds to an oil residue with lipids remaining on the vesicle after solvent drying and is also evident after five days, as shown in the red fluorescent image of Figure 7d. Monitoring the sample over time proved that the batch was fairly stable after 5 days resulting from a comparison between the GV size, as shown in Figure 7b,d.

In comparison with the work of Bao et al., we demonstrated that, even with a lower concentration (since we filtered the initial 15 mg/mL lipid solution), we could obtain rapid de-wetting by simply tuning the flow rates. Moreover, we maintain that our approach is more convenient because it is certainly more practical and faster to modify the flow rates with the same starting lipid solutions to find the ideal conditions of de-wetting rather than performing more experiments to change the concentrations of the solutions every time.

## 4. Conclusions

Microfluidics has been demonstrated to generate giant vesicles (GVs) of lipids that are able to maximize material encapsulation. By using an approach based on the production of Water-in-Oil-in-Water (W/O/W) double emulsions, followed by spontaneous octanol-assisted de-wetting, GVs with high size homogeneity and stability have been produced. The present work elucidates the importance of two aspects of microfluidic devices, which are the microchannel’s design and the fabrication material. We created a glass six-channel flow focusing (FF) junction geometry for the preparation of W/O/W double emulsions. By taking advantage of this design and of the physicochemical properties of the glass, it was found that our device can be used with a wide range of solvents without the functionalization of any microchannel walls, which is strictly necessary for polymeric microfluidic chips. Another benefit was the possibility to repeatedly exploit the same glass chip after easily cleaning the microchannels. All of these advantages entail reductions in costs and times for the device’s fabrication and ensure a certain reproducibility in the sample preparation that may not be guaranteed in the presence of a coating due to possible leaching and damage.

Furthermore, the hydrophilicity of glass and, on the other hand, the peculiarity of double hydrodynamic FF in a single junction enabled the generation of W/O/W double emulsions in a single step. The combination of these two features represents an innovation compared to glass or polymeric “tandem systems” and double FF junction in the same device and concentric glass capillaries for W/O/W generation. A water core was encapsulated in an octanol shell containing lipids, which, in turn, was simultaneously surrounded by a continuous aqueous phase. Solvent de-wetting induced the lipids to stabilize in a bilayer around the aqueous core, thus generating a GV. We demonstrated that, by fixing the starting lipids concentration, both the de-wetting process and the vesicle size are tunable depending on the relative flow rates of the three phases (internal water, oil with lipids, and external water) in the microchannels. 

We believe that our results establish a step forward for the microfluidic production of double emulsions and GVs and, above all, will help progress the synthetic biology field given the great interest in the employment of GVs as artificial cells.

## Data Availability

The original contributions presented in the study are included in the article, further inquiries can be directed to the corresponding author.
